# Beneficial Effect of Isoniazid Preventive Therapy and Antiretroviral Therapy on the Incidence of Tuberculosis in People Living with HIV in Ethiopia

**DOI:** 10.1371/journal.pone.0104557

**Published:** 2014-08-08

**Authors:** Kesetebirhan Delele Yirdaw, Degu Jerene, Zewdu Gashu, M. E. Edginton, Ajay M. V. Kumar, Yohannes Letamo, Beniam Feleke, Alula M. Teklu, Solomon Zewdu, Bill Weiss, Andrea Ruff

**Affiliations:** 1 Clinical Department, Johns Hopkins University TSEHAI Project, Addis Ababa, Ethiopia; 2 Center for Operational Research, International Union against Tuberculosis and Lung Disease, Paris, France; 3 International Union against Tuberculosis and Lung Disease, South-East Asia Regional Office, New Delhi, India; 4 Health Research and Technology Transfer Support Process, Southern Nations Nationalities and People's Region Health Bureau, Hawassa, Southern Nations Nationalities and People's Region, Ethiopia; 5 Care and treatment branch, TB/HIV unit, Centers for Disease Control and Prevention, Addis Ababa, Ethiopia; 6 PCM Department, Johns Hopkins University TSEHAI Project, Addis Ababa, Ethiopia; 7 Bloomberg School of Public Health, Johns Hopkins University, Baltimore, Maryland, United States of America; Royal Melbourne Hospital, Australia

## Abstract

**Background:**

IPT with or without concomitant administration of ART is a proven intervention to prevent tuberculosis among PLHIV. However, there are few data on the routine implementation of this intervention and its effectiveness in settings with limited resources.

**Objectives:**

To measure the level of uptake and effectiveness of IPT in reducing tuberculosis incidence in a cohort of PLHIV enrolled into HIV care between 2007 and 2010 in five hospitals in southern Ethiopia.

**Methods:**

A retrospective cohort analysis of electronic patient database was done. The independent effects of no intervention, “IPT-only,” “IPT-before-ART,” “IPT-and-ART started simultaneously,” “ART-only,” and “IPT-after-ART” on TB incidence were measured. Cox-proportional hazards regression was used to assess association of treatment categories with TB incidence.

**Results:**

Of 7,097 patients, 867 were excluded because they were transferred-in; a further 823 (12%) were excluded from the study because they were either identified to have TB through screening (292 patients) or were on TB treatment (531). Among the remaining 5,407 patients observed, IPT had been initiated for 39% of eligible patients. Children, male sex, advanced disease, and those in Pre-ART were less likely to be initiated on IPT. The overall TB incidence was 2.6 per 100 person-years. As compared to those with no intervention, use of “IPT-only” (aHR = 0.36, 95% CI = 0.19–0.66) and “ART-only” (aHR = 0.32, 95% CI = 0.24–0.43) were associated with significant reduction in TB incidence rate. Combining ART and IPT had a more profound effect. Starting IPT-before-ART (aHR = 0.18, 95% CI = 0.08–0.42) or simultaneously with ART (aHR = 0.20, 95% CI = 0.10–0.42) provided further reduction of TB at ∼80%.

**Conclusions:**

IPT was found to be effective in reducing TB incidence, independently and with concomitant ART, under programme conditions in resource-limited settings. The level of IPT provision and effectiveness in reducing TB was encouraging in the study setting. Scaling up and strengthening IPT service in addition to ART can have beneficial effect in reducing TB burden among PLHIV in settings with high TB/HIV burden.

## Introduction

The TB/HIV syndemic is a global public health challenge accounting for nearly 25% of all HIV-associated deaths [1]. Of 8.7 million estimated incident tuberculosis (TB) cases in 2011, about 13% were among people living with HIV (PLHIV) [1]. The African region, with 80% of the estimated HIV-infected TB cases, bears the brunt of the epidemic [1]. Ethiopia has the dual distinction of being a high TB burden country with incidence of 277 TB cases per 100,000 people per year [2] as well as a high HIV burden country with adult (15–49) HIV prevalence of 1.5% [3]. The prevalence of HIV among TB patients was estimated at 8% [1].

To reduce the burden of TB among PLHIV, the World Health Organization (WHO) recommends Intensified Case Finding (ICF), Isoniazid Preventive Therapy (IPT), Infection control, and early initiation of antiretroviral therapy (ART) [4]. Of these, ART is the most potent and widely implemented TB preventive intervention among PLHIV [4]. Although anti-retroviral drugs lower the risk of TB through immune reconstitution, the risk remains much higher than in HIV-uninfected individuals despite achievement of good CD4 cell recovery, emphasizing the need to implement other preventive interventions such as IPT. Observational studies done in Brazil and South Africa have shown that the combined effect of ART and IPT in preventing TB among PLHIV is significantly higher compared to using only ART [5]–[7]. Despite strong recommendations globally, the uptake of IPT has been limited due to difficulties in excluding active TB, added pill burden for patients, side effects, poor adherence to IPT, and concerns about development of drug resistance. In a review done by the WHO guidelines group, adherence for IPT ranged from 34–98% [4].

One study in Ethiopia demonstrated the effectiveness of IPT in reducing TB burden among PLHIV [8]. But, there is no published evidence on uptake and completion rates of IPT among PLHIV in Ethiopia, or its effectiveness in reducing TB incidence in combination with ART, when implemented under routine programmatic settings. There are also uncertainties regarding timing of IPT initiation in relation to ART. The WHO has identified these issues as priority areas for operational research [4]. In this study, we assessed the uptake and effectiveness of IPT in reducing TB incidence, when used alone or in combination with ART and the effect of timing of initiation of IPT in relation to initiation of ART in a population of PLHIV in Southern Ethiopia.

## Methods

### Ethics Considerations

Approval was obtained from the National Research Ethics Review Committee of Ethiopia. Furthermore, the original data collection for the pre-existing, de-identified/de-linked datasets used for this study was given a non-research determination and subsequent approval for secondary analysis by IRBs of Centers for Disease Control and Prevention (CDC) and Johns Hopkins University School of Public Health (JHSPH). Additional approval was obtained from the Ethics Advisory Group of the International Union against Tuberculosis and Lung Disease (IUATLD). Informed consent was not obtained since data was de-identified/de-linked before analysis.

### Study Design and Setting

This is a cohort study based on a secondary analysis of an electronic database of patients under care at five HIV/ART clinics in the Southern Nations, Nationalities and People's Region (SNNPR).The SNNPR, south of the Ethiopian capital Addis Ababa, has a population of about 17 million [9]. According to the latest Demographic and Health Survey of Ethiopia, the HIV prevalence for the region was estimated at 0.9% in 2011 [3]. Health services in the region are provided by 14 governmental, six non- government hospitals and 180 health centers. All public hospitals have provided ART and IPT for eligible patients based on the national ART and TB/HIV guidelines since 2005 and 2008 respectively. Eligibility for ART was based on the following criteria: all WHO stage 4 clients, WHO stage 3 clients with CD4≤350 and WHO stage 1 or 2 with CD4≤200. In the absence of CD4 testing, WHO stages 3 and 4 were eligible for ART. The first line ARV regimens used were according to national and international guidelines [10].

At each visit all HIV infected clients were screened for TB, using WHO recommended symptom-based screening, and symptomatic patients were evaluated by diagnostic tests (sputum smear microscopy, chest radiography, clinical evaluation) and with treatment provided where indicated [11]. Clients screening negative for TB and without contraindications were eligible to start IPT at any time. Tuberculin skin test (TST) was not done for patients before IPT initiation. IPT was provided for a period of 6 months at a dosage of 300 mg/day for adults and 5–10 mg/kg/day for children [11]. Patients collected their drugs every month until the treatment course was completed. Patients on IPT were monitored monthly for development of active TB, adherence to treatment and adverse drug reactions. All patient details were documented by trained and supervised health care workers, standardized to maintain accuracy [10], [12] and all services were provided free to the patients.

### Study Period and Population

The sampling frame constituted all 20 hospitals in SNNP region. All PLHIV newly enrolled in chronic HIV care in five randomly selected hospitals of SNNP region of Ethiopia between September 2007 and August 2010 constituted the study population. All cases of prevalent TB (TB cases already on treatment at the time of enrolment into HIV care and those diagnosed within one month of enrolment) were excluded from the cohort.

### Variables and Source of Data

The primary outcome variable was ‘incident TB’, defined as any new TB case that was diagnosed after one month of enrolment into HIV care. The primary exposures of interest were initiation of IPT and/or ART. We extracted data on these and other key covariates from the existing electronic database which included baseline WHO clinical stage, baseline CD4 count, age, sex, history of cotrimoxazole use, IPT completion and duration of ART intake. The data correspond to data on national forms for care of patients with HIV. The data were collected to support clinical decision-making and reporting to the government. All patients enrolling in HIV care at the clinic were registered in the database. Demographic information and other key milestones, like date of enrollment to care, date of ART initiation, date of IPT initiation, date of patient visit, and date of tuberculosis treatment initiation were recorded for each patient.

### Data Analysis

Epi Info statistical software Version 3.4 (United States Centers for Disease Control and Prevention, Atlanta, GA) and MS Access 2007 were used to clean and analyze the data. Person-time incidence rates, incidence rate ratios and hazard ratios were calculated using Stata statistical software Version 12. Person-time at risk of TB was accrued from the date of enrolment in chronic care until death, transfer to other facility, loss to follow-up (LTFU), appearance of incident TB, or last visit. Any patient over four weeks late for a scheduled appointment who failed to return to the ART programme and could not be located by “case managers” was classified as LTFU. The following two variables were treated as time-updated variables: IPT intake, with patients accruing person time in IPT groups after time of IPT initiation; ART intake, with patients accruing person time in ART groups after time of ART initiation. Based on this, patients were divided into six exposure categories to assess the combined effect of IPT intake, and ART treatment. ‘No intervention’ period when neither IPT nor ART were initiated; ‘IPT only’ period when IPT was the only treatment received; ‘ART only’ period when ART was the only treatment received; ‘IPT before ART’ period when ART was taken after IPT initiation; ‘IPT & ART simultaneously’ when IPT was initiated within two weeks of ART initiation; and ‘IPT after ART’ period when IPT was taken after ART initiation’. A more elaborate pictorial description of this time splitting process is found in figure S1. Similar procedure was followed to assess the overall effect of IPT completion and duration of ART treatment.

Missing values for baseline CD4 (22%) and WHO stage (10%) were calculated by multiple imputation methods using multivariate normal (mvn) regression. Twenty five data sets were generated. Site, year of enrolment, sex, age, cotrimoxazole use, ART status, INH use, and incident TB were used as regular variables to impute them. Survival methods were used to assess the relationship of independent variables listed above to incident TB. Participants were censored from the risk set after the first new TB diagnosis - multiple outcomes were not allowed. We fit a multivariable Cox proportional hazards regression model. Stratification was used to correct for effect of within-group correlation at site level on the relationship between independent and dependent variables. We constructed the model using stepwise techniques, adding one independent variable at a time to the model. The decision rule for addition of a variable to the model was if significance was less than 0.05, and the rule for removal was at significance more than 0.1. *P* values for risk ratios were calculated using chi-square test.

A secondary analysis was done taking the definition of incident TB as that occurring two months after enrolment. Finally, sensitivity analysis was done to assess if baseline characteristic of patients not on follow-up differed from those on follow-up and to see the effect of IPT on TB incidence, assuming 25% of randomly selected patients who were lost to follow-up or dead developed TB at time of censoring. The final dataset and program codes to generate the results of the study are presented as supporting information S1.

## Results

### Baseline Characteristics

**Figure 1:**
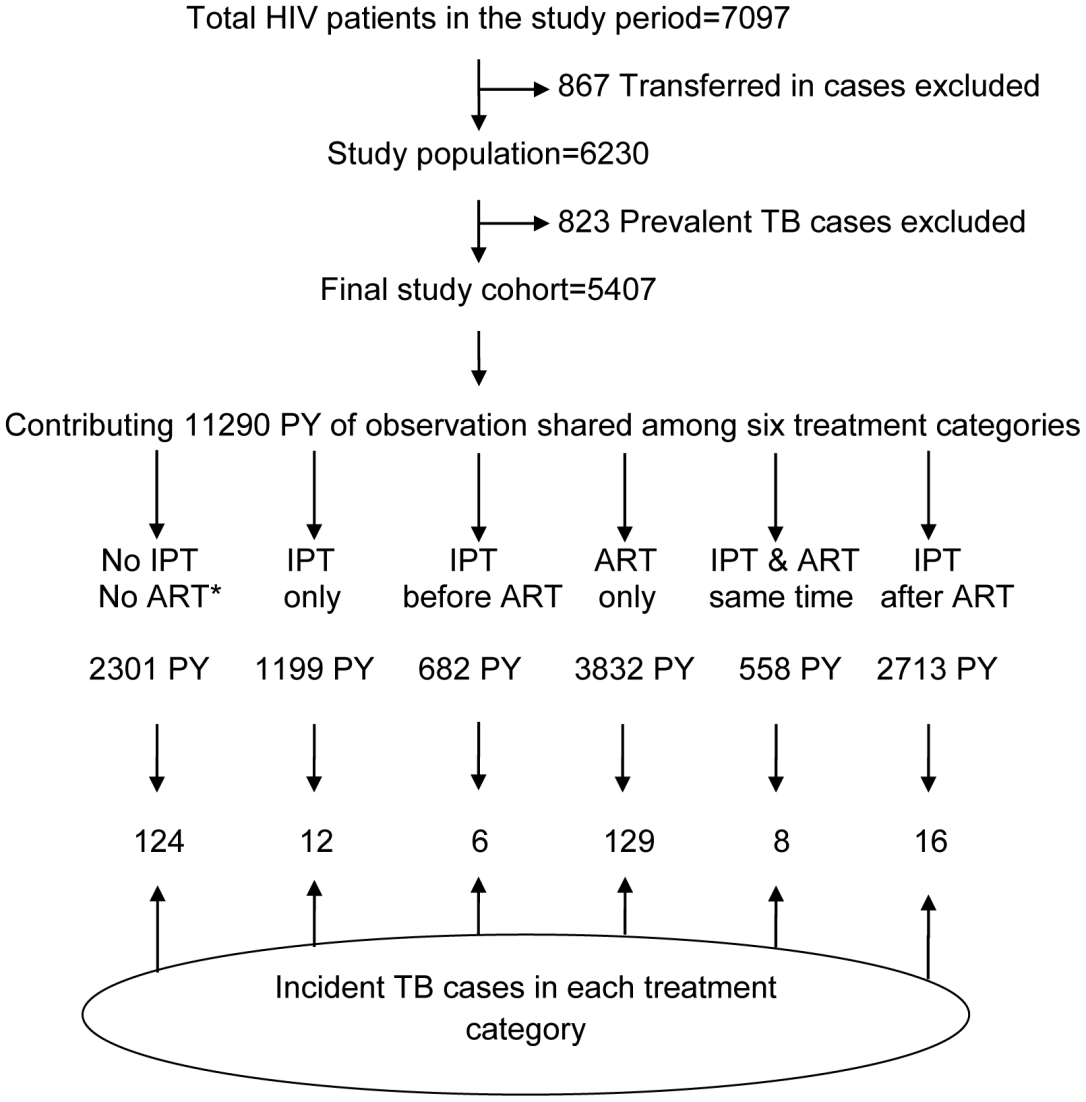
Cohort profile of study population, SNNP region, Ethiopia, September 2007 to August 2010. Of 7,097 patients enrolled in chronic HIV care, 5,407 were eligible for analysis contributing a total of 11,290 PY of follow-up. These were further classified into six treatment categories based on the combination of treatment received, IPT and/or ART, as well as the timing of IPT initiation with respect to ART. There were 295 incident TB cases diagnosed in the study period. IPT-Isoniazid Preventive Therapy; ART-antiretroviral therapy; TB-Tuberculosis; HIV-Human Immunodeficiency virus; PY-person year. * ‘No IPT NO ART’ group is equivalent to no intervention group.

Of the 7097 patients, 867 were excluded because they were transferred in; a further 823 (12%) were excluded from the study because they were either identified to have TB through screening (292 patients) or were on TB treatment (531). Thus, there were 5407 patients studied. (Figure 1)

Median age was 30 years (range, 0.16–82) and the median follow-up duration was two years with inter-quartile range (IQR) of 0.2–4 years. Most of the patients were adults more than 14 years of age (91%) and the proportion of females was 57%. (Table 1) Among patients on ART, patients with higher baseline CD4 count, and WHO disease stage 1 or 2 were more likely to have active follow-up at the end of the study (data not shown). Median baseline CD4 count was lowest for the ‘ART only’ group (152/mm^3^) followed by ‘IPT after ART’ (160/mm^3^), and ‘IPT and ART Simultaneously’ (187/mm^3^). Higher baseline CD4 counts were recorded for the ‘IPT only’ group (375/mm^3^) followed by ‘IPT before ART’ groups (242/mm^3^) and ‘no intervention’ group (216/mm^3^).

**Table 1:** Characteristics of HIV infected patients in chronic HIV care in SNNP region, Ethiopia, September 2007 to August 2010.

Category	Sub-category	Number (%)
**Age (years)**	<15	475 (9)
	15–50	4683 (87)
	>50	249 (5)
**Sex**	Female	3095 (57)
	Male	2312 (43)
**WHO Stage**	Stage 1 & 2	3182 (59)
	Stage 3 & 4	2225 (41)
**CPT initiation**	Yes	3926 (73)
	No	1481 (27)
**Baseline CD4 (count/mm^3^)**	<100	1002 (19)
	100–199	1545 (29)
	200–349	1590 (29)
	≥350	1270 (23)
**IPT intake**	Yes	2131 (39)
	No	3276 (61)
**Timing of IPT initiation** *	Before ART initiation	939 (44)
	After ART initiation	1192 (56)
**ART initiation**	Yes	3183 (59)
	No	2224 (41)

*among those who initiated IPT (n = 2131); IPT-Isoniazid Preventive Therapy; ART-antiretroviral therapy; CPT-Cotrimoxazole Preventive Therapy; WHO-World Health Organization; HIV-Human Immunodeficiency virus.

### IPT Initiation and Completion

IPT was initiated for 2131 (39%) patients, and 44% of them initiated IPT during Pre-ART care (Tables 1 and 2). Adults, women, those having CD4 count more than 100, those with earlier WHO disease stage and those having initiated ART were more likely to receive IPT. There was documented completion of IPT in 24% of those who started treatment. There were no statistically significant associations between IPT completion and other covariates.

**Table 2:** Characteristics of HIV infected patients in Chronic HIV care who took IPT in SNNP region, Ethiopia, September 2007 to August 2010.

Category	Sub-category	Total	Took IPT, n (%)	RR (95% CI)	*P* value
**Total**		5407	2131 (39)		
**Age (years)**	<15	475	125 (26)	1.0	
	15–50	4683	1898 (41)	**1.54 (1.34–1.77)**	**<0.001**
	>50	249	108 (43)	**1.65 (1.34–2.03)**	**<0.001**
**Sex**	Female	3095	1279 (41)	1.0	
	Male	2312	852 (37)	**0.89 (0.83–0.95)**	**<0.001**
**WHO Stage**	Stage 1 and 2	3182	1318 (41)	1.0	
	Stage 3 and 4	2225	813 (37)	**0.88 (0.82–0.94)**	**<0.001**
**CPT initiation**	Yes	3926	1751 (45)	1.0	
	No	1481	380 (26)	**0.58 (0.52–0.63)**	**<0.001**
**Baseline CD4 (count/mm^3^)**	<100	1002	345 (34)	1.0	
	100–199	1545	635 (41)	**1.19 (1.08–1.33)**	**0.001**
	200–349	1590	638 (40)	**1.17 (1.05–1.29)**	**0.004**
	≥350	1270	513 (40)	**1.17 (1.05–1.31)**	**0.004**
**ART initiation**	Yes	3183	1606 (50)	**1.0**	
	No	2224	525 (24)	**0.47 (0.43–0.51)**	**<0.001**

IPT-Isoniazid Preventive Therapy; ART-antiretroviral therapy; CPT-Cotrimoxazole Preventive Therapy; WHO-World Health Organization; HIV-Human Immunodeficiency virus.

### Incident Tuberculosis

There were 295 incident TB cases in this study cohort during 11,290 person-years (PY) of observation, making the overall TB incidence rate 2.6 per 100 PY of follow-up (95% CI: 2.3–2.9) (Table 3). The TB incidence rate in those who took IPT was 0.7 per 100 PY and 6.1 per 100 PY for those who did not (IRR: 0.11; 95% CI: 0.08–0.15 before controlling for effect of ART). This protective effect of IPT intake persisted whether IPT completion was documented or not.

**Table 3:** Incidence rate of TB among HIV infected patients in Chronic HIV care in SNNP region, Ethiopia, September 2007 to August 2010.

Category	Sub-category	Incident TB cases	Peron-years	Incidence rate per 100 person-years	Unadjusted HR (95% CI)	Adjusted HR (95% CI)
**Total**		295	11290	2.6		
**Age (years)**	<15	23	1099	2.1	1	
	15–49	253	9624	2.6	1.24 (0.81–1.91)	
	>49	19	568	3.3	1.61 (0.87–2.95)	
**Sex**	Female	139	6705	2.1	1	1
	Male	156	4585	3.4	**1.57 (1.25–1.97)**	**1.42 (1.13–1.79)**
**CPT status**	No	16	1546	1.0	1	
	Yes	279	9745	2.9	**3.16 (1.90–5.23)**	**2.15 (1.25–3.69)**
**WHO Stage**	Stage 1 or 2	74	6468	1.1	1	1
	Stage 3 or 4	221	4822	4.6	**4.05 (3.11–5.28)**	**3.80 (2.87–5,04)**
**Baseline CD4**	<100	93	2017	4.6	1	1
	100–199	82	3537	2.3	**0.54 (0.40–0.73)**	**0.70 (0.52–0.95)**
	201–349	84	3343	2.5	**0.59 (0.44–0.80)**	**0.59 (0.43–0.81)**
	>349	36	2394	1.5	**0.32 (0.22–0.47)**	**0.36 (0.23–0.56)**
**IPT Intake & completion** *	No IPT	244	3969	6.1	1	1
	IPT Completion	16	1975	0.8	**0.14 (0.08–0.23)**	**0.15 (0.09–0.26)**
	IPT non-completion	35	5347	0.7	**0.12 (0.08–0.17)**	**0.13 (0.09–0.19)**
**Duration on ART** *	No ART	136	3505	3.9	1	1
	0–5 months	76	952	8.0	0.90 (0.65–1.25)	**0.38 (0.27–0.54)**
	6–24 months	45	2868	1.6	0.76 (0.49–1.19)	**0.30 (0.19–0.48)**
	25–42 months	25	2519	1.0	**0.43 (0.23–0.82)**	**0.15 (0.08–0.28)**
	>42 months	13	1445	0.9	**0.37 (0.14–0.95)**	**0.10 (0.04–0.27)**
**IPT and/or ART** *	No intervention	124	2306	5.4	1	1
	IPT only	12	1199	1.0	**0.24 (0.13–0.44)**	**0.36 (0.19–0.66)**
	IPT before ART	6	682	0.9	**0.25 (0.11–0.59)**	**0.18 (0.08–0.42)**
	ART only	129	3832	3.4	**0.74 (0.58–0.96)**	**0.32 (0.24–0.43)**
	IPT & ART Simultaneously	8	558	1.4	**0.36 (0.17–0.74)**	**0.20 (0.10–0.42)**
	IPT after ART	16	2713	0.6	**0.19 (0.11–0.34)**	**0.08 (0.05–0.15)**

*Controlled for sex, WHO stage, baseline CD4, and Cotrimoxazole status; HR-Hazard Ratio; CI- Confidence Interval; IPT-Isoniazid Preventive Therapy; ART-antiretroviral therapy; CPT-Cotrimoxazole Preventive Therapy; WHO-World Health Organization; HIV-Human Immunodeficiency virus.

The first six months on ART represented the period with the highest risk (TB incidence 8.0 per 100 PY; 95% CI: 6.0–10), followed by Pre-ART period (TB incidence 3.9 per 100 PY; 95% CI: 3.3–4.6). After the first 6 months it decreased progressively with increase in length of time on ART. TB incidence was higher among ‘ART only’ and ‘No intervention’ treatment categories as compared to ‘IPT only’, ‘IPT before ART’, ‘IPT & ART simultaneously’ or ‘IPT after ART’. Patients who took IPT and were eventually started on ART or those who started both simultaneously had lower risk of developing TB as compared to those who took ART only. The combined effect of IPT and ART when started at the same time reduced TB incidence by 57% (IRR: 0.43; 95%CI 0.18–0.86) as compared to those who took ART only (Table 3).

### Cox Proportional Hazards Modeling

During bivariable analysis, male sex, cotrimoxazole use, advanced disease stage, and having a lower CD4 count were associated with increased relative hazard for developing TB (Table 3). On the other hand, being on IPT (irrespective of completion status), and taking ART for longer duration were each associated with lower relative hazard of developing TB. After controlling for sex, WHO stage, baseline CD4, and cotrimoxazole use, ART intake was protective against TB, and more so with greater duration of ART as compared to those not on ART. In bivariable analysis, all treatment categories showed protective effect but it was higher for ‘IPT only’ (HR 0.24, 95% CI 0.13–0.44), ‘IPT before ART’ (0.25, 95% CI 0.11–0.59), ‘IPT & ART simultaneously’ (HR 0.36; 95% CI 0.17–0.74) and ‘IPT after ART’ (HR 0.19, 95% CI 0.11–0.34). After controlling for sex, WHO stage, baseline CD4, and cotrimoxazole use, the ‘ART only’ group (aHR 0.32, 95% CI 0.24–0.43) had higher protective effect as well.

In the ‘IPT after ART’ group, TB incidence in the first six months after ART initiation was 0.2 per 100 PY as compared to those who were started on ART only which was at 6.2 per 100 PY. The median time for IPT initiation for the ‘IPT after ART’ group was nine months (IQR-3 months to 1.6 years).

Hazard ratios were calculated taking the definition of incident TB to be new TB disease diagnosed after two months from enrolment and the results were similar to those of the original analysis. Sensitivity analysis was also done taking those started on ART and assuming 25% of those lost to follow-up or dead developed TB. IPT was still protective of TB (Table 4). The percentages of patients lost to follow-up or dead among ‘IPT before ART’, ‘ART only’, ‘IPT and ART simultaneously’, and ‘IPT after ART’ treatment groups were 9%, 39%, 17%, and 9% respectively. The overall lost or death among ART patients was 24%.

**Table 4:** Sensitivity analysis for effectiveness of IPT with or without ART using Cox proportional hazards model among patients on ART assuming those who were lost to follow-up and dead developed TB in SNNP region, Ethiopia, September 2007 to August 2010.

Category	Sub-category	Incident TB+lost+dead	Person-years	Incidence rate per 100 person-years	Unadjusted HR (95% CI)	Adjusted HR (95% CI)
**IPT and/or ART** *	No intervention	93	1018	9.1	1	1
	IPT only	9	370	2.4	**0.36 (0.18–0.71)**	**0.40 (0.20–0.80)**
	IPT before ART	15	682	2.2	**0.42 (0.24–0.74)**	**0.45 (0.25–0.80)**
	ART only	266	3832	6.9	0.93 (0.73–1.19)	**0.69 (0.53–0.89)**
	IPT & ART Simultaneously	14	558	2.5	**0.39 (0.22–0.70)**	**0.35 (0.20–0.62)**
	IPT after ART	41	2713	1.5	**0.30 (0.20–0.46)**	**0.22 (0.15–0.34)**

*Controlled for sex, WHO stage, cotrimoxazole use, and baseline CD4; HR-Hazard Ratio; CI- Confidence Interval; IPT-Isoniazid Preventive Therapy; ART-antiretroviral therapy; WHO-World Health Organization; HIV-Human Immunodeficiency virus.

## Discussion

Over a third of PLHIV received IPT and its use was associated with 65% reduction in tuberculosis occurrence in patients who had not yet started ART. On the other hand, using only ART was associated with 68% reduction in TB incidence. The effect of ART use was directly proportional to the duration of treatment. Despite that, TB incidence among patients on ART for more than three and a half years remained about four times as high as the current estimate for the general population of Ethiopia which is 240 per 100,000 per year [2]. Combining IPT and ART resulted in further reduction in TB incidence, reaffirming the synergistic role of the two interventions. Timing of IPT initiation relative to ART initiation did not make any difference in reduction of TB incidence. Taking IPT before starting ART was associated with 82% reduction in TB incidence while starting IPT & ART simultaneously resulted in 80% reduction. TB incidence was much higher during the first six months of ART suggesting a possibility of TB immune reconstitution inflammatory syndrome (IRIS) which occurs in the face of very low CD4 count [13]. In the study cohort, 40% of TB cases diagnosed in this period had CD4 below 100, supporting the aforementioned premise.

The protective effect of ART and IPT was similar to what was previously reported in Ethiopia [8], [14].The protective effect obtained from combining ART and IPT including the beneficial effect of starting IPT before ART is well documented in the studies done in South Africa and Brazil [5], [6]. Significant TB incidence reduction among patients who started IPT and ART simultaneously was a new finding.

The protective effect of initiating IPT after starting ART, which was at 92%, should not be confused with this being the best option to prevent TB in the first six months. This group of patients was actually low risk for TB to begin with. TB incidence for this group prior to IPT initiation but after ART was 0.2 per 100 PY as compared to those who took ART only at 6.2 per 100 PY. Patients in the ‘IPT after ART’ group survived initial treatment period without experiencing TB and thus were likely to have been healthier overall. So, a policy offering IPT after ART initiation and after the risky period of TB has passed allows those at risk for it to develop TB disease and would benefit only the minority who were not at risk for TB in the first place. What would be rational is to initiate IPT before ART if possible or simultaneously with ART. This would benefit all patients but particularly those at risk for TB IRIS in the first six months. Of course, IPT should be offered if for some reason it was not initiated during these periods because it would further decrease TB.

TST-positive patients benefit the most from IPT as compared to those who are TST- negative [4]. In studies where TST status was not determined results were mixed with some showing protective effect and others not [4]. Ethiopia is a high TB burden country with TST positivity among the general population reaching up to 66% [4], [15]. In this study TST status was not determined, yet the protective effect of IPT was comparable to those studies with positive TST status [4].

Patients ever started on cotrimoxazole were found to be at higher risk for TB. This is not surprising, considering that those with advanced immune-suppression who were eligible for it would have also been at risk for TB [4], [11].The association persisted even after controlling for other potential confounding variables.

This study has implications for policy and practice. We now have additional data on the effectiveness of IPT in preventing TB in routine care setting. A simple modeling indicates that, tripling the IPT coverage in two year time in the study region from the current level following the recommended IPT initiation would reduce the number of TB patients among PLHIV in care by 27% than if no more patients were initiated on IPT after this (Text S1). This should help convince health workers, patients, and policy makers in quickly addressing the IPT implementation challenges to harness its benefit [16]. The potential benefit of initiating IPT before ART or simultaneously with ART if coupled with effective implementation of TB screening could reduce TB burden in the first six months of ART intake. The IPT coverage in this cohort of HIV infected patients is over twice the national average of 18% for HIV infected patients in chronic care in Ethiopia [1], [17], [18] but the coverage rate was slightly lower in children. This suggests the need to look for and address additional underlying factors that lead to lower IPT use rate in the pediatric population in particular and the PLHIV in general. A study done in South Africa indicates that lack of knowledge and experience among providers about TB screening and IPT use as well as lack of awareness of benefits of IPT among patients contributed to low IPT use [19]. IPT use for those with advanced HIV disease was found to be lower as comparable to those with less advanced disease. But since the risk for TB is higher in this group, IPT use should have been relatively higher as well. Initiating more patients as such could be challenging due to higher rates of symptomatic patients in this group and the difficulty faced by health care workers in excluding active TB. Late presentation of patients to chronic HIV care adds to this burden [16]. This is another reminder for the need to have feasible, improved, and less expensive diagnostic methods in such settings.

There are several points that make the finding of this study important. The study showed the level to which IPT intervention was scaled up and the resultant effectiveness on reduction of TB risk. The study was done in routine clinical care setting in a low-income country and this will add to the limited evidence of IPT effectiveness under such conditions. A previous study done in Ethiopia on factors affecting occurrence of TB showed IPT use prevented TB (adjusted odds ratio 0.35). Similar assessments were done in Brazil and South Africa and comparable results were documented in the effectiveness of IPT with ART while IPT coverage (10 and 13% respectively) and effectiveness of ‘IPT only’ (43 and 13% respectively) were much lower than the findings from our study [5], [6]. In a different study in Tanzania, IPT was not found to be effective at all [20]. Our study presents new evidence on the effect of timing of IPT initiation with respect to ART. There are ongoing clinical trials looking into this which will clear uncertainties [21].

Although coverage of IPT for this cohort of HIV infected patients was lower than the expected, it was more than twice the prior estimate for the national average of 18% among HIV infected patients in chronic care [1], [17], [18]. The coverage is even higher for patients ever started on ART. It was also better than reported coverage in other African countries such as Mozambique (<1%); but lower than that of South Africa where it was 46% in 2011 [22], [23]. The relatively better IPT coverage rate in this cohort could be attributed to the technical support provided by JHU TSEHAI, a PEPFAR-supported program, which involved monthly site-level mentoring, training health care providers, IPT-focused update sessions, and improved data capturing system.

Long-term ART was associated with greater reduction of risk of TB in this study. The result is comparable to a study done in South Africa that found that TB risk decreased significantly with long-term ART treatment although it remained at four times the risk to the general population [7]. Findings of higher TB incidence in the early months of ART in this study were similar to findings from a large multinational cohort study in six sub-Saharan African countries with TB incidence in the first three months being 13 per 100 PY, a bit higher than TB incidence in Pre-ART period at 10.5 per 100 PY [24].

Our findings should be interpreted cautiously because of some important limitations. Missing data and incomplete documentation were the major limitations for some variables. Missing data for baseline CD4 and WHO stage were accounted for by imputation. IPT completion was documented in 24% of the records only, which did not allow for thorough comparison on the effect of IPT by adherence rate. Being on ART was associated with higher completion of IPT in Brazil [24], for example, but this was not demonstrated in the present study. Also, because of the limited number of variables in the database, we were not able to include variables such as anemia and body-mass index in our analyses. TB diagnosis was presumptive or was based on sputum and chest x-ray results in most cases, which might have led to over or under estimation of the actual number of TB cases. Patients who were lost to follow-up or died had lower CD4 counts and more advanced WHO disease stage putting them at greater risk for TB as compared to those active in follow-up. Hence, TB incidence was likely to be a bit higher in this study population.

The strengths of this study include that it was adequately powered to study the relationship between the effect of ART used alone and with IPT in reducing TB because of large sample size and adequate number of events. Since all study records were included the potential for selection bias was reduced.

## Conclusions

This study indicates that IPT intake in addition to ART could be beneficial in reducing risk of TB among PLHIV in high TB and HIV setting in resource-limited countries. The most benefit would be harnessed if IPT was initiated before or at the same time with ART. The gap in IPT coverage should be addressed urgently and in particular children less than 14 years with HIV that represent a group at significant risk for TB must be prioritized. Further studies are needed to assess the level of adherence, and barriers to IPT provision.

## Supporting Information

Figure S1 **Splitting of subjects according to time-updated.** Coding of time updated variables: ART = 0 means ART not received; ART = 1 means ART received; IPT = 0 means IPT not received; IPT = 1 means IPT received; Subject 1 received no intervention for nine years for which reason value of both ART and IPT was set to ‘0’ for the whole duration. Subject 2 received no intervention for the first three years but after that IPT was received and stayed in care for seven years. So the value of IPT was updated to ‘1’ in the period where IPT was received. Subject 3 received no intervention for the first three years but after that ART was received and stayed in care for six years. So the value of ART was updated to ‘1’ in the period where ART was received. Subject 4 received no intervention for the first four years, took IPT for three years followed by initiation of ART. In the period ART was initiated, this subjected was exposed for the effects of both ART and IPT for three years but with the effect of IPT coming first. So the value of IPT was updated to ‘1’ in the period where IPT only was received, then after ART initiation, both values of ART and IPT was made to be ‘1’. Subject 5 experienced the same chain of events as in Subject 4, except ART initiation preceded IPT initiation. Subject 6 received no intervention for the first five years followed by initiation of both ART and IPT at the same time and stayed in care for five years. For this reason, value of ART and IPT was updated to ‘1’ at the same time. Subjects 2–5 contributed person-time of follow-up to more than one treatment category.
(TIF)

Text S1 **Estimated effect of IPT scale-up on incidence of TB/HIV co-infection.**
(DOCX)

Supporting Information S1 **Dataset and program codes.**
(ZIP)
