# Supplementary figures and images for: Beneficial Effect of Isoniazid Preventive Therapy and Antiretroviral Therapy on the Incidence of Tuberculosis in People Living with HIV in Ethiopia
Source: PLoS One. 2014 Aug 8;9(8):e104557. doi: 10.1371/journal.pone.0104557 (PMC4126726; doi:10.1371/journal.pone.0104557)

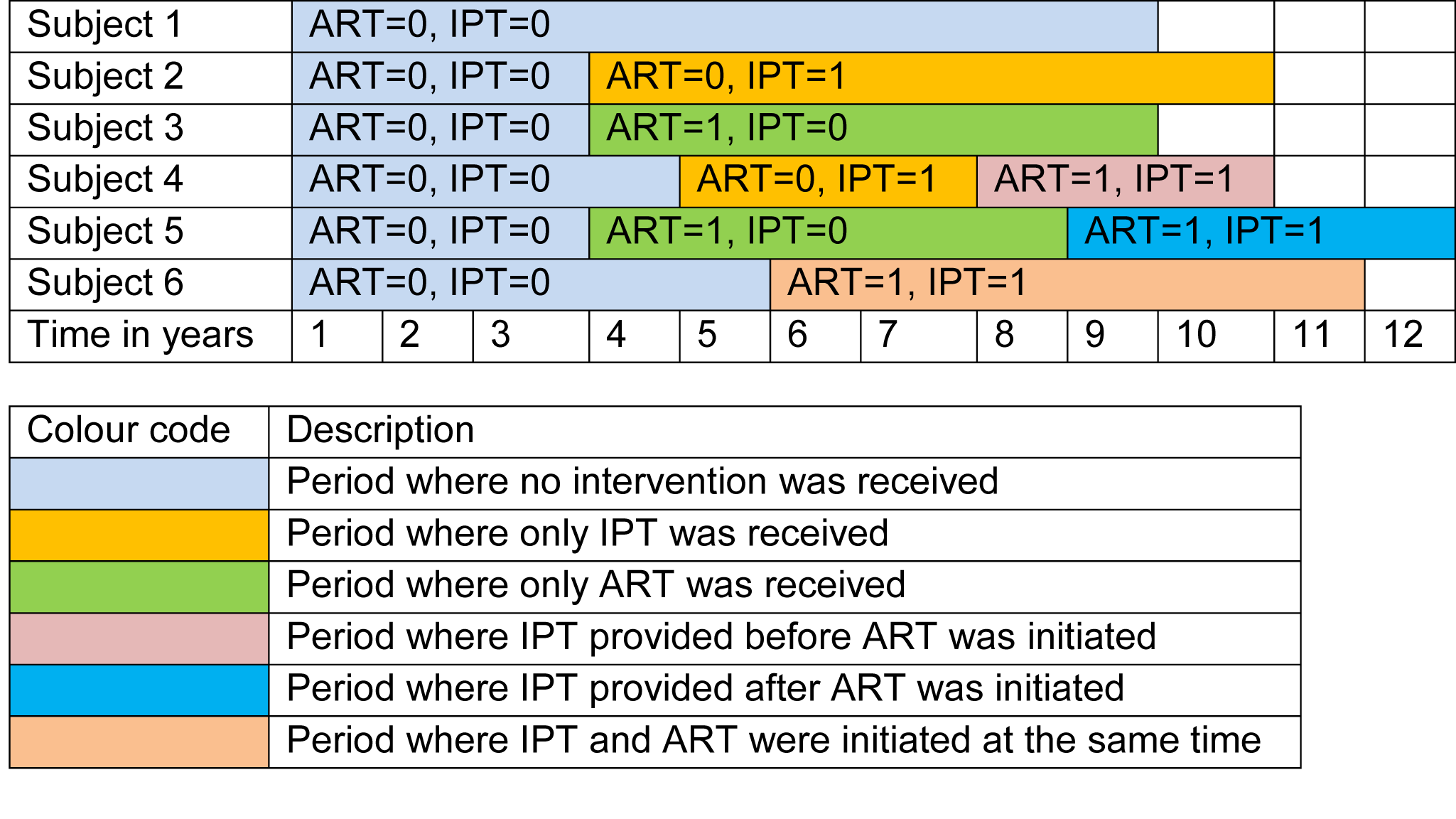

Supplement: Figure S1 — Splitting of subjects according to time-updated. Coding of time updated variables: ART = 0 means ART not received; ART = 1 means ART received; IPT = 0 means IPT not received; IPT = 1 means IPT received; Subject 1 received no intervention for nine years for which reason value of both ART and IPT was set to ‘0’ for the whole duration. Subject 2 received no intervention for the first three years but after that IPT was received and stayed in care for seven years. So the value of IPT was updated to ‘1’ in the period where IPT was received. Subject 3 received no intervention for the first three years but after that ART was received and stayed in care for six years. So the value of ART was updated to ‘1’ in the period where ART was received. Subject 4 received no intervention for the first four years, took IPT for three years followed by initiation of ART. In the period ART was initiated, this subjected was exposed for the effects of both ART and IPT for three years but with the effect of IPT coming first. So the value of IPT was updated to ‘1’ in the period where IPT only was received, then after ART initiation, both values of ART and IPT was made to be ‘1’. Subject 5 experienced the same chain of events as in Subject 4, except ART initiation preceded IPT initiation. Subject 6 received no intervention for the first five years followed by initiation of both ART and IPT at the same time and stayed in care for five years. For this reason, value of ART and IPT was updated to ‘1’ at the same time. Subjects 2–5 contributed person-time of follow-up to more than one treatment category. (TIF) [file pone.0104557.s001.tif]
