# Supplementary material for: Beneficial Effect of Isoniazid Preventive Therapy and Antiretroviral Therapy on the Incidence of Tuberculosis in People Living with HIV in Ethiopia
Source: PLoS One. 2014 Aug 8;9(8):e104557. doi: 10.1371/journal.pone.0104557 (PMC4126726; doi:10.1371/journal.pone.0104557)
Supplement: Text S1 — Estimated effect of IPT scale-up on incidence of TB/HIV co-infection. (DOCX) [file pone.0104557.s002.docx]

**Text S1: Estimated effect of IPT scale-up on incidence of TB/HIV co-infection**

Current status:

- Total number of patients active on follow-up=35000
- Current IPT coverage=20%
- Number of patients who took IPT=35000x20%=7000
- Proportion of patients who took IPT and on ART=60%
- Number of patients who took IPT and on ART=7000x60%=4200
- Number of patients who took IPT and on PreART=7000-4200=2800
- PLHIV currently on ART=28000
- PLHIV active in Pre ART=7000

Scenario 1: Maintaining status quo of IPT coverage for the coming two years

- Total number of patients active on follow-up=46000 (assuming 7000 ART and 4500 Pre ART patients add to the existing pool)
- Number of patients who took IPT for the coming 2 years unchanged =7000
- Number of patients who took IPT and on ART=4200
- Number of patients who took IPT and on Pre ART=2800
- PLHIV on ART after 2 years=35000;
  - Risk of TB for those on ART but without IPT=3.4 per 100 person-year
  - Number of new TB patients expected per year among those already on ART but without IPT = (35000-4200) x 0.034=1047
  - Risk of TB for those on ART and IPT=0.6 per 100 person-year
  - Number of new TB patients expected per year among those on ART and IPT = 4200 x 0.006=25

→ Total number of new TB patients expected with current IPT coverage among those in ART= 1072

- PLHIV active in Pre ART after 2 years =11500;
  - Risk of TB for those on Pre-ART but without IPT=5.4 per 100 person-year
  - Number of new TB patients expected per year among those in Pre-ART but without IPT = (11500-2800) x 0.054=470
  - Risk of TB for those in Pre ART and IPT=1.0 per 100 person-year
  - Number of new TB patients expected per year among those on ART and IPT = 2800 x 0.01=28

→ Total number of new TB patients expected with current IPT coverage among those in Pre ART= 498

→ Total number of TB patients expected with current IPT coverage among those in active HIV care = 1072+498=1570

Scenario 2: Increasing IPT coverage for the coming two years by 200% to 21000

- Total number of patients active on follow-up=46000 (assuming 7000 ART and 4500 Pre ART patients add to the existing pool)
- Number of patients on IPT after 2 years increased to =21000
- Number of patients who took IPT and on ART=14000 (assuming IPT for all newly initiated ART patients, and 2800 patients already on ART in addition to the existing 4200 who took IPT and ART)
- Number of patients who took IPT and on Pre ART=7000 (assuming IPT for 4200 new PreART patients put on IPT in addition to the 2800 ones already in PreART)
- PLHIV on ART after 2 years=35000;
  - Risk of TB for those on ART but without IPT=3.4 per 100 person-year
  - Number of new TB patients expected per year among those already on ART but without IPT= (35000-14000) x 0.034=714
  - Risk of TB for those already on ART and IPT=0.6 per 100 person-year
  - Number of new TB patients expected per year among those on ART and IPT = 7000 x 0.006=42
  - Risk of TB for those newly started on ART and IPT=1.4 per 100 person-year
  - Number of new TB patients expected per year among those on ART and IPT= 7000 x 0.014=98

→ Total number of new TB patients expected with increased IPT coverage among those in ART= 714+42+98=854

- PLHIV active in Pre ART after 2 years =11500;
  - Risk of TB for those on Pre-ART and without IPT=5.4 per 100 person-year
  - Number of new TB patients expected per year among those in Pre-ART and without IPT = (11500-7000) x 0.054=243
  - Risk of TB for those in Pre ART and IPT=0.1 per 100 person-year
  - Number of new TB patients expected per year among those on ART and IPT = 7000 x 0.001=70

→ Total number of new TB patients expected with current IPT coverage among those in Pre ART= 313

→ Total number of TB patients expected with current IPT coverage among those in active HIV care = 854+313=1167. This is a 26% reduction of TB incidence in Scenario 2 as compared to Scenario 1. In other words, if hypothetically, the same number of patients were seen per month as that in Scenario 1 (1570÷12=131), then number of new TB patients in Scenario 2 would be exhausted in 1167÷131≈9 months, meaning three months free of TB/HIV co-infection.
