# Supplementary material for: Beneficial Effect of Isoniazid Preventive Therapy and Antiretroviral Therapy on the Incidence of Tuberculosis in People Living with HIV in Ethiopia
Source: PLoS One. 2014 Aug 8;9(8):e104557. doi: 10.1371/journal.pone.0104557 (PMC4126726; doi:10.1371/journal.pone.0104557)
Supplement: Supporting Information S1 — Dataset and program codes. (ZIP) [file pone.0104557.s003.zip › IPT Data/Read me.docx]

Follow the following simple steps exactly to replicate outputs of this study as presented in the tables.

Step 1:

Unzip the contents of this folder onto your local disc (C:)

Step 2:

Then, open your Stat statistical software and run the ‘DO files’ located in the ‘Analysis codes’ folder one by one. There are text explanations of what happens when every code runs.

Step 3:

After running a DO file, make sure you delete contents of the ‘Temp’ folder (and not the folder itself) which stores temporary data sets created during analysis, before running it or another DO file again.

N.B.:

- Don’t change names, location or content of any of the files in order to guarantee you get the output right.
- Since records are randomly selected every time you run the code for sensitivity analysis, you may get slightly different output every time you run it.
